# Effects of isometric leg training on ambulatory blood pressure and morning blood pressure surge in young normotensive men and women

**DOI:** 10.1038/s41598-021-04092-z

**Published:** 2022-01-10

**Authors:** Anthony W. Baross, Robert D. Brook, Anthony D. Kay, Reuben Howden, Ebony C. Gaillard, Ben D. H. Gordon, Kevin J. Milne, Cheri L. M. McGowan, Ian L. Swaine

**Affiliations:** 1 Sport and Exercise Science, University of Northampton, University Drive, NN1 5PH Northampton, UK; 2 Division of Cardiovascular Diseases, Wayne State University, Detroit, MI USA; 3 Laboratory of Systems Physiology: Department of Applied Physiology, Health and Clinical Sciences, UNC Charlotte, Charlotte, NC USA; 4 Department of Exercise and Rehabilitative Sciences, Slippery Rock University, Slippery Rock, PA USA; 5 Department of Kinesiology, University of Windsor, Windsor, Canada; 6 Sport Science, University of Greenwich, London, UK; 7 Sport and Exercise Physiology, University of Northampton, University Drive, Northampton, NN1 5PH UK

**Keywords:** Physiology, Diseases

## Abstract

Despite the reported association between diurnal variations in ambulatory blood pressure (BP) and elevated cardiovascular disease risk, little is known regarding the effects of isometric resistance training (IRT), a practical BP-lowering intervention, on ambulatory BP and morning BP surge (MBPS). Thus, we investigated whether (i) IRT causes reductions in ambulatory BP and MBPS, in young normotensives, and (ii) if there are any sex differences in these changes. Twenty normotensive individuals (mean 24-h SBP = 121 ± 7, DBP = 67 ± 6 mmHg) undertook 10-weeks of bilateral-leg IRT (4 × 2-min/2-min rest, at 20% maximum voluntary contraction (MVC) 3 days/week). Ambulatory BP and MBPS (mean systolic BP (SBP) 2 h after waking minus the lowest sleeping 1 h mean SBP) was measures pre- and post-training. There were significant reductions in 24-h ambulatory SBP in men (− 4 ± 2 mmHg, *P* = 0.0001) and women (− 4 ± 2 mmHg, *P* = 0.0001) following IRT. Significant reductions were also observed in MBPS (− 6 ± 8 mmHg, *p* = 0.044; − 6 ± 7 mmHg, *P* = 0.019), yet there were no significant differences between men and women in these changes, and 24-h ambulatory diastolic BP remained unchanged. Furthermore, a significant correlation was identified between the magnitude of the change in MBPS and the magnitude of changes in the mean 2-h SBP after waking for both men and women (men, r = 0.89, *P* = 0.001; women, r = 0.74, *P* = 0.014). These findings add further support to the idea that IRT, as practical lifestyle intervention, is effective in significantly lowering ambulatory SBP and MBPS and might reduce the incidence of adverse cardiovascular events that often occur in the morning.

## Introduction

Cardiovascular disease (CVD) is a major cause of mortality and responsible for approximately one-third of deaths globally each year^1^. The World Health Organisation (WHO) estimates that the global health crisis associated with hypertension, a modifiable risk factor for CVD including coronary heart disease, peripheral arterial disease and strokes^2^, will reach epidemic levels in the near future^1^. Additionally, there is a well-established diurnal variation in the onset of adverse cardiovascular events with elevated incidence of myocardial infarction, strokes^3^ and atherosclerotic plaque rupture^4^ upon waking and during early morning (6 am 10 am) compared to other times of day. It is believed that this diurnal variation of adverse cardiovascular events may be linked with altered physiological parameters including reduced baroreceptor sensitivity, increased neuro-humoral and sympathetic nerve activity which are also thought to follow a circadian pattern^2,4–6^.

Twenty four-hour (24-h) ambulatory blood pressure (BP) has been shown to have a greater association with cardiovascular events than the more common clinical (office-based) resting BP^7,8^. In particular, diurnal BP variability is considered to be associated with increased CVD risk. Morning BP surge (MBPS) is a normal occurrence associated with the diurnal variation in BP over a 24-h period resulting from a drop in BP during sleep and a surge in the morning upon waking. Hypertensive individuals, due to their higher daytime BP, appear to have elevated MBPS compared to their normotensive counterparts^3^. Furthermore, elevated MBPS (~ 4–6 h after waking) is considered a significant cardiovascular risk factor for both hypertensive^3,9,10^ and normotensive [3,10,] individuals and may predict adverse cardiovascular events^10^. More specifically, elevated MBPS is thought to be associated with increased stroke risk (independent of 24-h BP) and to be a destabilising factor for atherosclerotic plaque^2,11^. Therefore, exaggerated MBPS in normotensive and hypertensive individuals may be a trigger for the higher frequency of early morning adverse cardiovascular events.

Isometric resistance training (IRT) was recently endorsed by the American College of Cardiology/American Heart Association as one of the “best proven nonpharmacological interventions for prevention and treatment of hypertension” in their most recent guidelines^12^. Commonly undertaken 3 times per week (4 × 2-min isometric contractions per session) for 8–10 weeks at moderate exercise intensities (20–30% maximum voluntary contraction, MVC), IRT is most often performed using a portable handgrip dynamometer, or with a lower body (leg extension) dynamometer. IRT is an effective method of lowering resting systolic BP (SBP; − 5 to − 10 mmHg) and diastolic BP (DBP; − 3 to − 5 mmHg) following either upper^13–17^, or lower body^13,17–19^ protocols. More recently, IRT has been reported to reduce 24-h ambulatory (− 4 mmHg), daytime (− 3 to − 4 mmHg) and night-time (− 3 to − 4 mmHg) BP in both normotensive men and women^17^. Previous studies have reported no significant differences in the magnitude of these BP reductions between men and women^17^.

To date, few studies have investigated the effects of IRT on ambulatory BP or diurnal BP variability and none have investigated its effects on the morning surge in BP in normotensive or hypertensive individuals. Therefore, the purpose of this study was to determine whether (i) IRT causes reductions in ambulatory BP and MBPS, in young normotensives and (ii) there is sexual dimorphism in these changes. Therefore, we hypothesised that there will be a significant change in diurnal BP variability and MBPS in young normotensive men and women following 10 weeks of IRT.

## Materials and methods

### Participants

**Figure 1:**
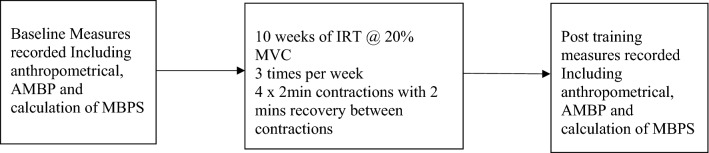
Summary of the IRT protocol and pre/post measures.

Following ethical approval from the University of Northampton’s Ethics review panel, twenty normotensive (< 140/ < 90 mmHg), apparently healthy individuals were recruited to the study (10 men, mean ± SD: age 21 ± 4 years, height 182 ± 6 cm, mass 82 ± 6 kg; 10 women, age 23 ± 5 years, height 157 ± 2 cm, mass 65 ± 15 kg). Participants were recreationally active (IPAQ), undertaking at least 2 but no more than 4 exercise sessions per week, were non-smokers (including vaping and cannabis) and were not prescribed medication. All participants received a participant information sheet explaining in detail the experimental protocol and any risks involved, then completed and signed an informed consent and pre-test medical questionnaire to determine eligibility, thereby adhering to the guidelines set by the 1964 Declaration of Helsinki. If still interested and eligible, participants were then familiarised with the test protocols and measures. Following baseline data collection, participants undertook 10 weeks of supervised isometric leg extension (bilateral, ILE) training. Post-intervention testing took place in the week following the completion of the training intervention, no less than 48 and no more than 72 h after the final training session and within 2 h of baseline data collection time-of-day (see Fig. 1). Both pre- and post-training data collection sessions were undertaken by the same individual in a temperature-controlled environment (20–23 °C) at least two hours post-prandially. Participants abstained from caffeine in the 12 h prior to testing and did not consume alcohol, take over-the-counter medication or undertake vigorous exercise for 24 h preceding the data collection sessions. Both baseline and post-training measures in women were taken during the same phase of the menstrual cycle^14^.

### Protocol

#### Ambulatory blood pressure

Portable BP monitors (Meditech, P.M.S. Instruments Ltd. Maidenhead, Berks, UK), a validated monitor for measuring ambulatory BP^20^ were used to measure 24-h, daytime and night-time ambulatory BP at 30 min intervals during daytime, defined as the time participants rose until they retired to bed, and then hourly at night, defined as the time that participants went to bed, until they awoke^3,21,22^. 24-h recordings were only used when ≥ 80% valid measures were present and there were no missing hours of data^23,24^. Following the baseline ambulatory BP monitoring participants reported their everyday activities that they undertook during this period, including their sleep patterns, which were used to calculate MBPS. Subsequently, participants were asked to undertake similar activities and sleep patterns for the post-training assessment, to help standardise the BP measures.

#### Morning blood pressure surge calculation

There are several methods used to calculate MBPS including sleep-trough and pre-waking^2,3,10,21^. However, there is no consensus on a preferred method^2^. Additionally, when analysing MBPS previous studies have adjusted for various factors including 24-h ambulatory BP^10,25^ whilst others^25^ have noted that this adjustment should not occur as components of MBPS are also components of 24-h ambulatory BP. In this study sleep-trough MBPS was calculated as the mean of the four SBP readings in the 2-h period just after waking, minus the mean of the two readings centred around the lowest nocturnal SBP reading^3,21,22^ without adjusting for 24-h ambulatory BP.

#### Isometric resistance training

Participants undertook supervised, onsite ILE training three times per week for 10 weeks using an isokinetic dynamometer (Biodex Medical Systems Inc. New York, USA). Each session consisted of 4 × 2-min isometric contractions at 20% MVC (determined at the start of each training session), with 2-min rest periods between contractions. Each training session was separated by at least 24 h. Additionally, dietary, nutritional and exercise changes were monitored and recorded throughout the 10-week training programme, using personal logs.

### Statistical analysis

Data were assessed for normal distribution and presented as mean ± SD. Statistical analysis was performed using IBM SPSS Statistics 22 software (SPSS Inc., Chicago, Illinois, USA). All baseline ambulatory BP measures were assessed for differences between men and women using as one-way analysis of variance (ANOVA) with a Bonferroni correction post-hoc analysis. A two-way repeated measures ANOVA was used to assess the within and between groups ambulatory BP (mean 24-h, daytime, night time and diurnal variation) and MBPS. Pearson’s product moment correlation coefficient was used to assess the relationship between the magnitude of the change in MBPS and the magnitude of changes in daytime ambulatory SBP after IRT training. Statistical significance was set at *P* ≤ 0.05.

Effect size (Cohen’s d) was calculated from a previous IRT study employing similar interventions from mean changes 24-h ambulatory BP^17^. To ensure adequate statistical power for all analyses was conducted using the following parameters (power = 0.80, alpha = 0.05, men, effect size = 0.9, women, effect size = 0.89). The calculated sample size required for statistical power was 10 for both groups.

## Results

### Reliability

The day-to-day reliability of the ambulatory BP measures was determined by calculating the intraclass correlation coefficients (ICC) and coefficient of variation (expressed as a percentage on the mean). For 24-h SBP, daytime SBP and night-time SBP no significant difference was detected between day-to-day mean values for any measure. The ICC values were 0.73, 0.71 and 0.81 and the coefficients of variation were 2.5%, 2.3%, and 2.6%, respectively.

All participants completed the thirty training sessions over the 10-week IRT programme. Baseline data (Table 1) indicates a significant difference between men and women for height and body mass, as expected. However, there were no other significant differences in baseline characteristics between sexes. Participants reported no changes in diet or exercise over the 10-week training programme. Additionally, no adverse effects of the training intervention or the data collection sessions were reported.

**Table 1:** Participant baseline data.

	Men (*n* = 10)	Women (*n* = 10)
Age (yrs)	21 ± 4	23 ± 5
Height (cm)	182 ± 6	157 ± 2*
Body mass (Kg)	82 ± 6	65 ± 15*
BMI (Kg/m^2^)	25 ± 1	26 ± 5
**Ambulatory blood pressure**		
24-h SBP (mmHg)	123 ± 8	119 ± 6
24-h DBP (mmHg)	66 ± 8	68 ± 5
Daytime SBP (mmHg)	127 ± 7	123 ± 5
Daytime DBP (mmHg)	69 ± 7	68 ± 5
Night-time SBP (mmHg)	107 ± 5	103 ± 5
Night-time DBP (mmHg)	53 ± 6	51 ± 4
Morning SBP (mmHg)	137 ± 4	134 ± 3
Lowest Night-time SBP (mmHg)	103 ± 6	101 ± 4
MBPS (mmHg)	34 ± 5	33 ± 5

Values are means ± SD. *BMI* body mass index, *SBP* systolic blood pressure, *DBP* diastolic blood pressure, *MBPS* morning blood pressure surge.

*Significantly different from men (*P* < 0.05).

#### Effects of isometric resistance training on ambulatory blood pressure

**Figure 2:**
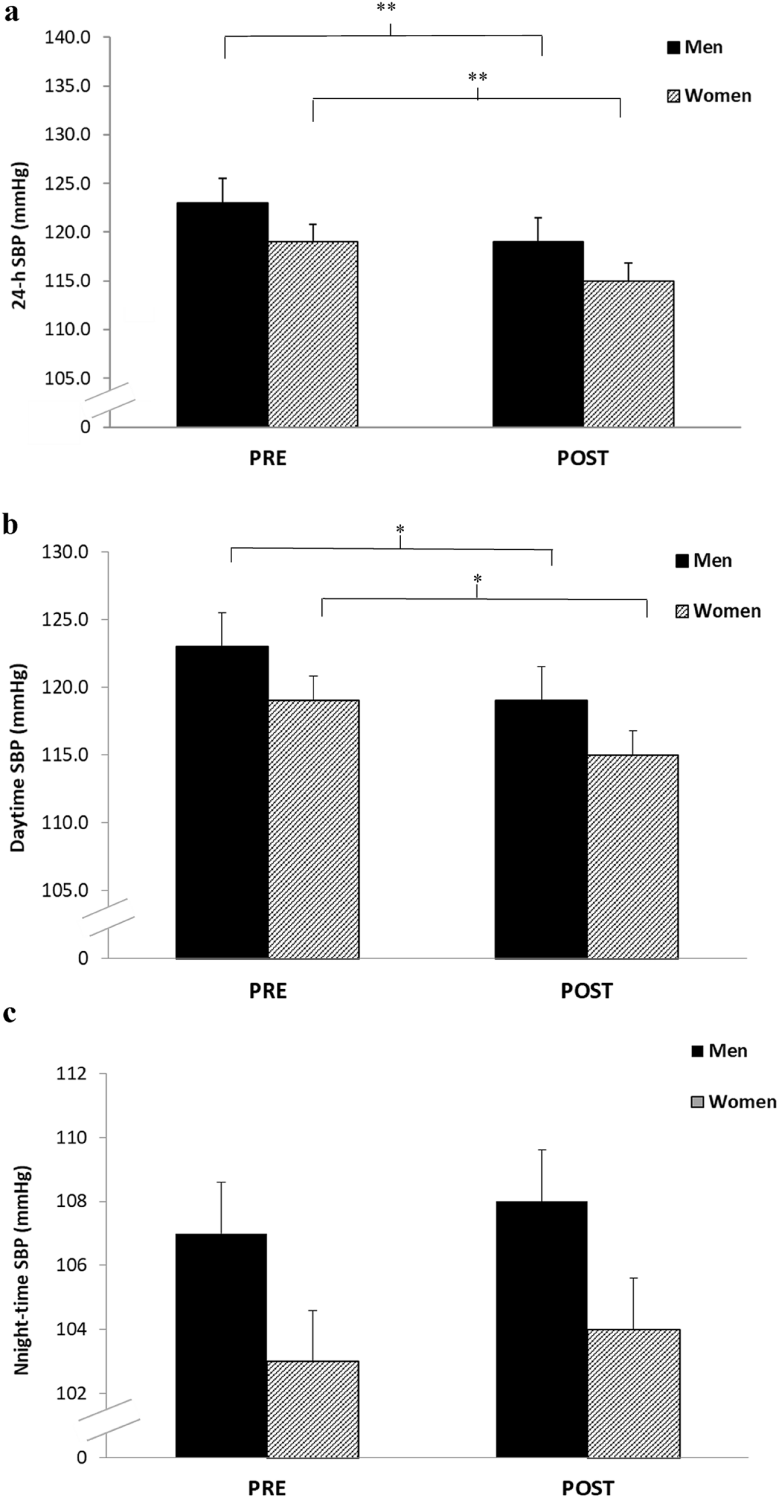
Effects of 10 weeks of isometric resistance training (IRT) on (**a**), 24-h (**b**), Daytime and (**c**), Night-time ambulatory systolic blood pressure (SBP) for men and women. *P value < 0.05, **P value < 0.01.

There were significant reductions in 24-h ambulatory SBP (− 4 ± 2 mmHg, *P* = 0.0001; − 4 ± 2 mmHg, *P* = 0.0001) in both men and women respectively following the 10-week IRT intervention. This comprised of a significant reduction in daytime SBP (− 5 ± 5 mmHg, *P* = 0.019; -5 ± 4 mmHg, *P* = 0.002) but not night time SBP (− 1 ± 5 mmHg, *P* = 0.75; − 1 ± 3 mmHg, *P* = 0.3, see Fig. 2). Statistical analysis of the magnitude of the changes, indicated no sex interactions for all ambulatory SBP measures (24-h, daytime and night time) following IRT. Additionally, although there were some observed changes in ambulatory DBP (Men: 24-h, 66 ± 8 to 64 ± 7 mmHg; daytime, 69 ± 7 to 66 ± 7 mmHg; night-time 53 ± 6 to 54 ± 7 mmHg; Women: 24-h, 68 ± 5 to 68 ± 4 mmHg; daytime, 68 ± 5 to 65 ± 6 mmHg; night-time 51 ± 4 to 51 ± 5 mmHg) over time, these changes were not significant.

#### Effects of isometric resistance training on morning blood pressure surge

**Figure 3:**
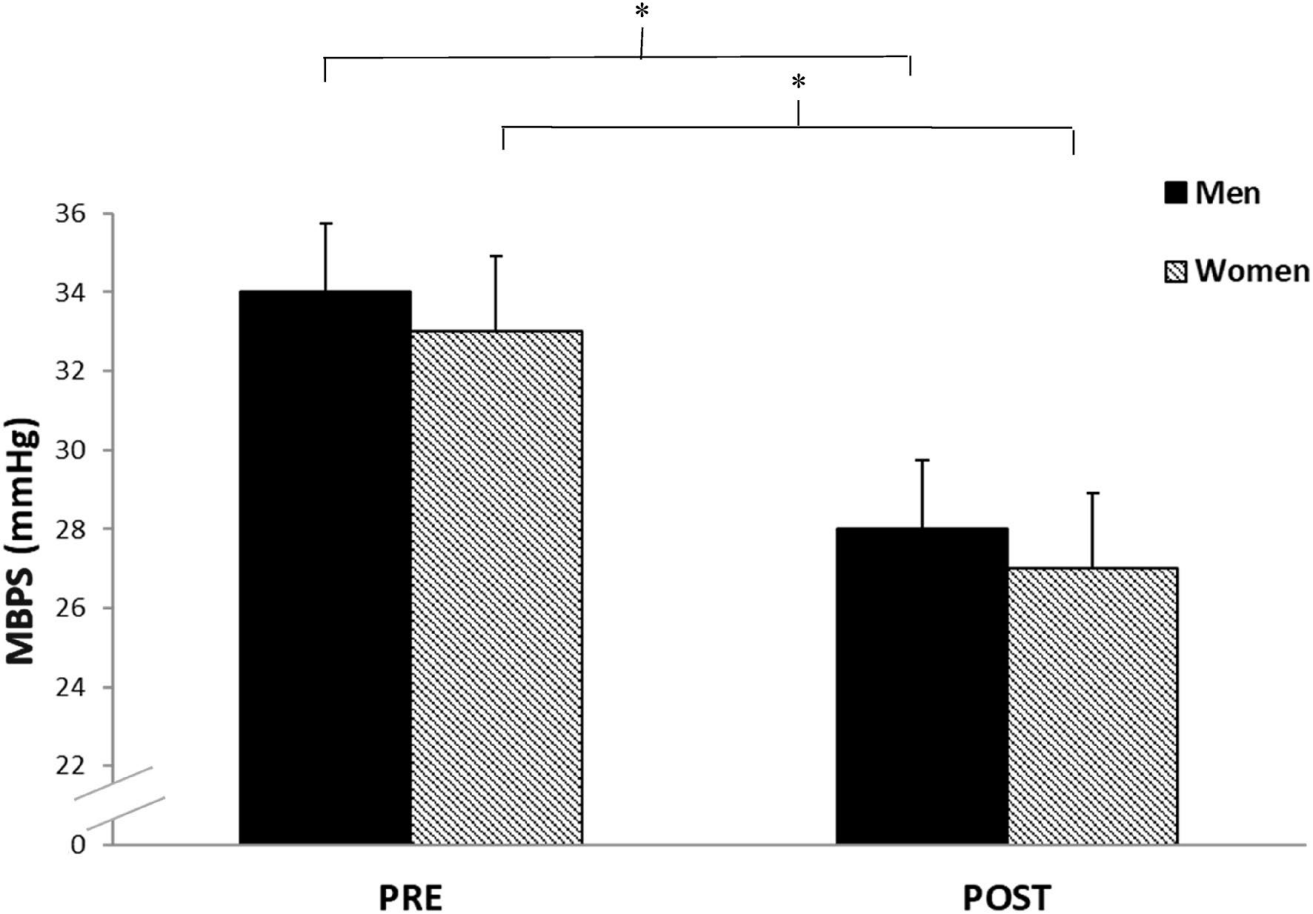
Effects of 10 weeks of isometric resistance training (IRT) on morning blood pressure surge (MBPS) for men and women. *P value < 0.05.

**Figure 4:**
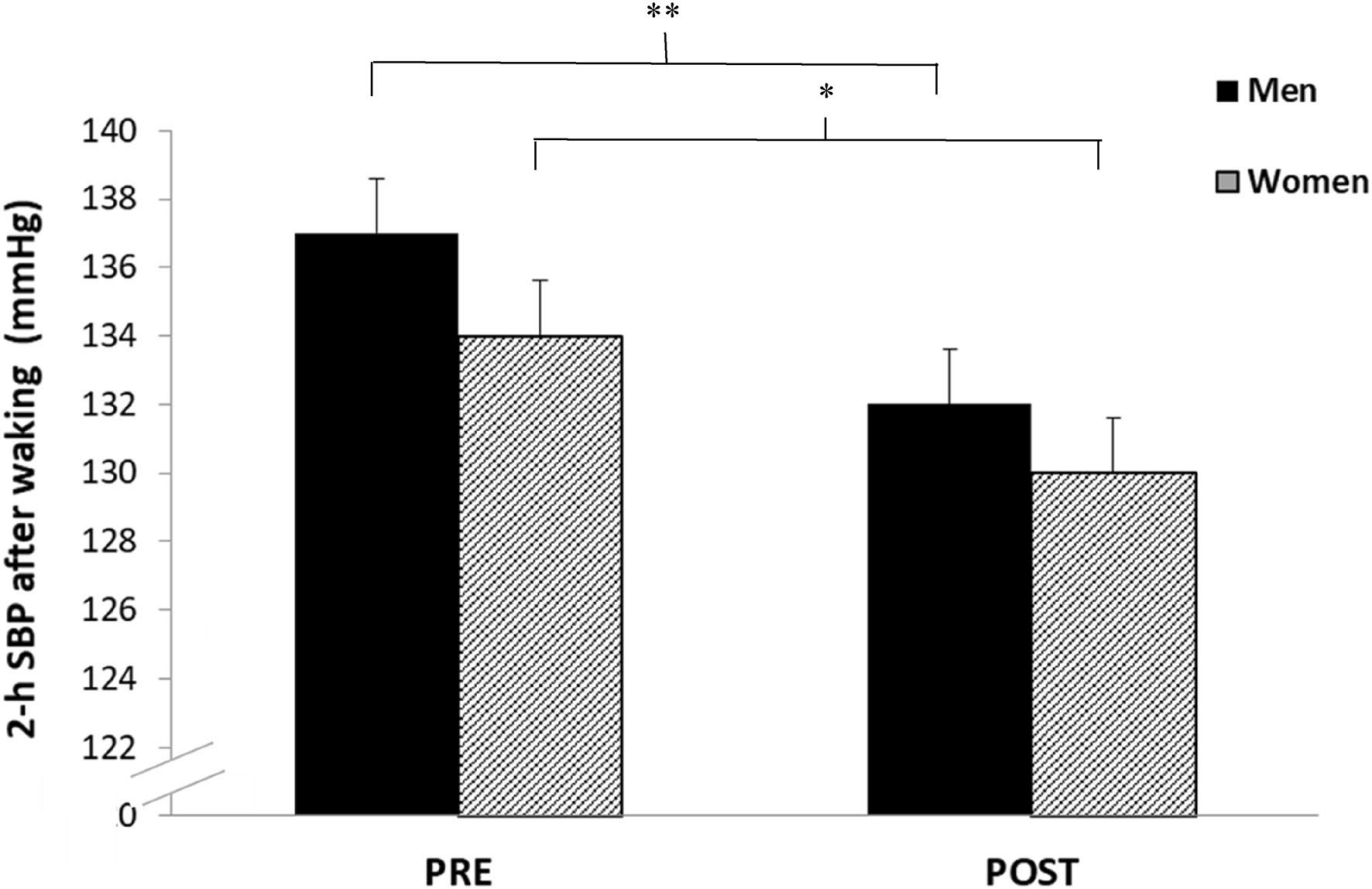
Effects of 10 weeks of isometric resistance training (IRT) on mean 2-h systolic blood pressure (SBP) after waking for men and women. *P value < 0.05, **P value < 0.001.

**Figure 5:**
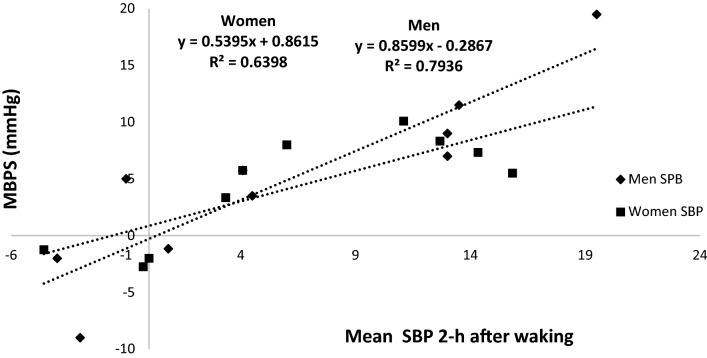
Regression line and plot of changes in morning blood pressure surge (MBPS) and changes in 2-h systolic blood pressure (BP) after waking following 10 weeks of isometric resistance training for men and women.

**Figure 6:**
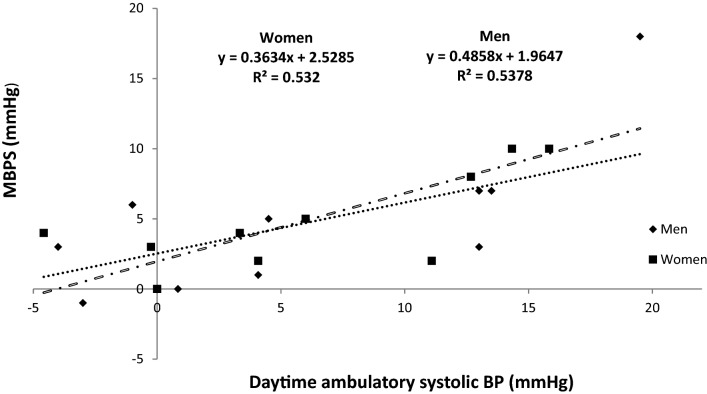
Regression line and plot of changes in morning blood pressure surge (MBPS) and changes in daytime ambulatory systolic blood pressure (SBP) following 10 weeks of isometric resistance training for men and women.

Analysis of the calculated MBPS demonstrated significant reductions (− 7 ± 5 mmHg, *P* = 0.044; − 7 ± 10 mmHg, *P* = 0.019, see Fig. 3), which was associated with a concomitant decrease in the mean SBP 2-h after waking (− 5 ± 4 mmHg, *P* = 0.002; − 4 ± 7 mmHg, *P* = 0.026, see Fig. 4) but not night-time SBP (1 ± 5 mmHg, *P* = 0.75; 1.1 ± 3.2 mmHg, *P* = 0.3) for both men and women, respectively. Furthermore, there were no significant differences in the magnitude of the changes in MBPS or the reduction in mean SBP 2-h after waking between men and women. Moreover, a significant correlation was identified between the magnitude of the change in MBPS and the magnitude of changes in mean SBP 2-h after waking for both men and women, such that greater reductions in mean SBP 2-h after waking were correlated with larger decreases in MBPS (men, r = 0.89, *P* = 0.001; women, r = 0.74, *P* = 0.014, see Fig. 5). However, apart from a significant correlation between the magnitude of the change in MBPS and the magnitude of change in daytime SBP (men, r = 0.73, P = 0.016; women, r = 0.73, *P* = 0.017, see Fig. 6) no further correlations were found for 24-h (men, r = 0.30, *P* = 0.039; women, r = 0.56, *P* = 0.087) or night time ambulatory SBP (men, r = 0.004, *P* = 0.99; women, r = 0.44, *P* = 0.199).

## Discussion

This study is the first to investigate the concomitant effects of IRT on diurnal BP variability and MBPS in young normotensive men and women. In addition to replicating previously-observed reductions in 24-h and daytime ambulatory BP following training^17^, we observed significant reductions in MBPS in addition to a strong correlation between the magnitude of the change in MBPS and changes in daytime ambulatory BP. Furthermore, as previously reported the changes in ambulatory BP^17^ and MBPS did not differ significantly between men and women.

The WHO^1^ have reported an increase in noncommunicable diseases, such as hypertension to near epidemic levels, and now consider hypertension prevention to be a key global health priority. Furthermore, as the prognosis of CVD and hypertension is thought to have a greater association with ambulatory BP rather than clinical BP, and MBPS is considered to be closely linked with increased cardiovascular events, these novel findings have important clinical implications and would indicate that IRT may be beneficial for the chronic management of BP in young healthy individuals. Most lifestyle treatments (e.g., aerobic exercise) are even more effective in hypertensives than normotensives. We posit therefore, that IRT will likely follow this same pattern. If these responses are confirmed in individuals with pre- and overt hypertension, IRT may prove especially effective for those diagnosed with hypertension or exaggerated MBPS (threshold levels between > 34 and > 55 mmHg)^3,10,21^.

The significant reductions in 24-h ambulatory BP (both men and women, − 4 mmHg) reported in this study following completion of the IRT intervention are comparable with those reported in previous IRT studies (both men and women, − 4 mmHg) as is the daytime ambulatory BP (men, − 5 mmHg; women, − 5 mmHg) compared to men, − 3 mmHg and women, − 4 mmHg^17^. In contrast, the present study did not find significant reductions in night time ambulatory BP where others have^17^ or have reported clinically relevant reductions^24^. However, the reductions in both daytime and night time ambulatory BP reported by Stiller-Moldovan et al.^24^ were observed in medicated hypertensive individuals.

The novel findings of this study demonstrate a significant reduction in MBPS (men, − 6 mmHg; women, − 6 mmHg) which have not been reported in IRT studies previously. To the authors’ knowledge there are no previous training intervention studies that have investigated the effects of exercise training, aerobic or resistance (or any combination), on changes to the observed morning surge in BP. However, previous studies reporting reductions in daytime systolic ambulatory BP with small or no drop in night time ambulatory BP may have produced an attenuated MBPS without reporting the change. The present study has also reported a significant correlation (men, r = 0.89; women, r = 0.79) between the magnitude of the change (Δ) in the mean ambulatory SBP 2-h after waking and the change in MBPS in addition to a significant correlation between Δ daytime SBP and Δ MBPS (men, r = 0.73; women, r = 0.73, without similar correlations between Δ night-time or 24-h ambulatory BP and Δ MBPS. Therefore, it seems that in this study at least, as MBPS was calculated using the lowest night-time SBP and daytime SBP 2 h after waking, the main attributing factor to the reduction in MBPS is the decrease in the daytime ambulatory SBP upon waking, without a concomitant drop in night time SBP.

The clinical implications of this observed reduction in MBPS are clear from the literature. Kario^2^ noted that treatments which reduced the surge in morning BP may provide significant benefit for hypertensive individuals. Additionally, it is thought that a reduced MBPS could reduce the incidence of adverse cardiovascular events such as myocardial infarction, stroke and atherosclerotic plaque rupture. The mechanism for this is likely to be related to the circadian variation in patterns such as vasoconstriction responses, flow distribution balance and elevated sheer stress reported in hypertensives^5^. However, this study did not include measures that would allow for the identification of the probable mechanism(s) responsible for the findings. This remains to be elucidated in future studies. Nevertheless, one of the main physiological factors, that has been proposed previously, which may have contributed to the observed surge in morning BP upon waking is the circadian elevation in sympathetic nervous system activity (SNA) in the morning^21^. The consequence of the elevated SNA in the morning, is increased vasoconstriction and hence elevated BP. It is thought that alongside the increased SNA, elevated arterial stiffness, cortisol and catecholamine levels could also play a role in the increased MBPS^21^.

SNA directed to the heart and skeletal muscle is elevated during exercise and this is likely to result in increased vasoconstriction of the vasculature surrounding both active and inactive musculature^26^. However, the elevation in SNA can be attenuated in the exercising muscle^25,26^. This reduced or inhibited sympathetically activated vasoconstriction is termed functional sympatholysis (FS)^26^. It is thought that exercise training, including IRT, could improve FS. Indeed, Mortensen et al.^26^ have shown that 8 weeks of aerobic exercise training improved FS. Furthermore, the same authors showed a link between aerobic training status and FS^26^. They also theorised that altered training induced α-adrenergic responsiveness associated with vasoconstriction could be strongly linked to the observed augmented FS^26^.

Although it is speculative to suggest that the observed changes in ambulatory BP and MBPS in this study could be explained by an improved FS, other research^27^ has reported that ischemic preconditioning (IPC; consisting of repeated bouts [5 min.] of unilateral ischemia followed by reperfusion) attenuated sympathetic vasoconstriction in healthy individuals. Although performed at a much higher intensity and duration, IPC is not dissimilar to the ischemic component of IRT. Furthermore, Kimura et al.^28^ observed improved blood flow and hence FS within the contralateral limb following IPC, suggesting a possible systemic effect. It is also thought that stimulation of ATP activated potassium channels (K_ATP_) may contribute to improved FS and this has previously been shown to be one of the effects of IPC exercise^3,27^. Therefore, it is plausible that repeated exposure to an ischemia–reperfusion stimulus using IRT may enhance FS via improved K_ATP_ channel activity. Although these purported mechanisms are speculative, at this time the data presented in this study suggest that IRT may improve vascular function which has been previously reported to be enhanced through improved FS^28,29^. However, additional research needs to be undertaken to further explore these possible mechanisms and the reported reductions seen in MBPS following IRT.

In summary, the concomitant decreases in 24-h and daytime systolic ambulatory BP in addition to the reduction in MBPS observed in the present study are important findings. The results support previous research showing that IRT is effective in lowering ambulatory BP. These findings may also have significant clinical implications because the significant reductions in the MBPS might reduce the incidence of adverse cardiovascular events, which often occur in the morning. The findings offer the potential for clinically meaningful CVD and stroke risk reduction, provided these effects can be demonstrated in those who are at risk.

This preliminary work is promising, although we do acknowledge the lack of a control group. Despite this limitation, we are confident that this work makes a novel contribution to the literature, and provides a solid platform for future investigations. To strengthen our confidence in these findings we have reported reliability data for the ambulatory SBP measures in the absence of a control group. We also emphasise that all participants were fully supported and supervised during the IRT period, their physical activity levels remained constant during the training period and all undertook similar activities and had similar sleep patterns during ambulatory BP measurements. These findings highlight the need for robust randomised clinical trials to establish the effectiveness of IRT in reducing the incidence of adverse cardiovascular events. Furthermore, such trials might compare effectiveness of different forms of exercise on 24-h ambulatory BP, morning blood pressure surge and incidence of adverse cardiovascular events. There is also a need to look more closely at the mechanisms associated with the changes reported within this study.
